# Optimal Test Design for Estimation of Mean Ability Growth

**DOI:** 10.1177/01466216241291233

**Published:** 2024-10-15

**Authors:** Jonas Bjermo

**Affiliations:** 1 Linköping University, Sweden; 2 7675 Stockholm University, Sweden

**Keywords:** ability growth, item response theory, optimal design, optimum in-average, particle swarm optimization, simulated annealing, test information

## Abstract

The design of an achievement test is crucial for many reasons. This article focuses on a population’s ability growth between school grades. We define design as the allocating of test items concerning the difficulties. The objective is to present an optimal test design method for estimating the mean and percentile ability growth with good precision. We use the asymptotic expression of the variance in terms of the test information. With that criterion for optimization, we propose to use particle swarm optimization to find the optimal design. The results show that the allocation of the item difficulties depends on item discrimination and the magnitude of the ability growth. The optimization function depends on the examinees’ abilities, hence, the value of the unknown mean ability growth. Therefore, we will also use an optimum in-average design and conclude that it is robust to uncertainty in the mean ability growth. A test is, in practice, assembled from items stored in an item pool with calibrated item parameters. Hence, we also perform a discrete optimization using simulated annealing and compare the results to the particle swarm optimization.

Measuring the ability growth between grades in school has been important for some time. It makes it possible to compare the progression of knowledge between groups such as schools or countries. It is desired to have good precision (low variance) when estimating ability growth.

When the objective is to estimate the ability of a single examinee with good precision, for example, under the 2PL IRT model, the best procedure is to select items with the difficulty parameter close to the true ability and with the discrimination parameter as high as possible. It is due to the form of the Fisher information for the 2PL model (see, e.g., Buyske, 2005). When the objective is to estimate some function of all the examinees’ abilities, it is not obvious how to choose the item difficulty parameters. We, therefore, need some further theory to derive the optimal design when the objective is to estimate a function of many examinees’ abilities.

Optimal design theory is customary for selecting, for example, persons to estimate parameters with good precision. Atkinsson et al. (2007) and Berger and Wong (2009) provide a good review of optimal design theory. One type of optimal design problem in educational testing is test design (or test assembly), which determines how to design a test depending on the purpose of the test. Birnbaum (1968) proposed to use the test information function (TIF) when constructing tests. The function TIF(*θ*) determines the expected Fisher information at some ability level *θ*. The TIF can have different shapes depending on the purpose of the test. It can have a uniform shape for an aptitude test or be high at a specific level for a certification test. Using item response theory, the shape of the TIF will determine the allocation of the test items regarding the difficulty of the items. For a further review of test design, see Buyske (2005), van der Linden (1998), and van der Linden (2005).

Optimal test design for efficiently estimating abilities using linear programming has been studied by, for example, Theunissen (1985), van der Linden and Boekkooi-Timminga (1989), Berger (1994), and Berger (1998). The latter examined the selection of item parameters for different distributions of the examinees’ abilities. Berger (1998) examined how to optimally select the item difficulty parameter *b* considering several separate ability distributions when keeping the discrimination parameters *a* constant at different levels. Berger (1998) used the D-optimality and maximin criterion as the optimal criterion and concluded that optimal item selection depends on the values of the discrimination parameter and the distribution of the examinees’ abilities. Berger (1998) also concluded that the difficulties of the items tend to follow the ability distribution when the values of the discrimination parameters increase.

We have seen that selecting items optimally for designing a single test has been investigated thoroughly in the literature. However, optimally selecting items of two tests with anchor items has not been completely examined. Bjermo and Miller (2021) examined, in a simulation study, the effect of item difficulty distribution on the precision of the estimated ability growth between groups. The study used two tests with common items and compared several ways to select items regarding difficulty. The same setup will be used but a statistical criterion method is proposed to determine the optimal selection of item difficulties. The items will be optimally allocated regarding the precision of the mean ability and percentile growth estimate. We are only considering a test design regarding the item difficulty parameter using an IRT model, which means we are not considering different test lengths, different numbers of anchor items, or content constraints of the test.

The focus of this study is the distance *d* = *μ*_2_ − *μ*_1_ between the mean of the ability distributions of the same population of examinees at different time points and the percentile difference *d*_*p*_ = *θ*_2*p*_ − *θ*_1*p*_ for the *p*th percentile. The estimate $\hat{d}$ is the estimated growth of mean ability from one point in time to another based on random samples from the distribution. The random samples are different for each time point even though the population is the same. The ability growth from one school grade to another is usually of interest in reality.

The main objective is to demonstrate a particle swarm optimization on a multi-objective weighted sum criterion to determine the optimal design for different scenarios and present the appurtenant results. The search space is the item difficulties of the two tests. The optimization algorithm stops when a pre-determined convergence criterion is fulfilled and provides an optimal solution. An optimal solution here means to fulfill the convergence criterion within a reasonable time and attain very similar solutions under repetition of the algorithm. The results will be presented from the proposed method for several different cases. The method can, of course, be used for several scenarios not covered here. A two-parametric logistic item response (2PL IRT) model will be assumed.

The optimal designs depend on the examinees’ abilities and hence the mean *μ* and the mean ability growth *d*, which is unknown beforehand. One way is to assume an initial distribution of the examinees’ abilities, but it might be very uncertain. Therefore, as a second objective of the study, we also consider an optimal design when uncertainty is present in *d*.

The particle swarm optimization on the multi-objective weighted sum criterion is an unrestricted optimization problem since the solution can take on any values within the pre-determined bounds. The item discrimination parameter *a* and item difficulty parameter *b* also come in pairs since they belong to every item pairwise. The parameters are, therefore, pairwise related. The unrestricted optimization will be supplemented with a restricted one where items are optimally selected from an item pool using the simulated annealing algorithm and compare the results.

We present values of the item parameter difficulties that produce the optimal solutions in several graphs for different values of *d*, *p*, and discrimination parameter *a*. The results show that the general picture is that the difficulty parameter is more spread out with increasing values of *a*. With increasing *d*, and when *a* > 1, the solution is more concentrated to specific values both for the unique and common items.

The study starts with defining the variance functions for the estimated difference of ability means and percentiles in terms of the Fisher information. We then describe how to discretize a continuous distribution to use the defined variance functions as objective functions when optimizing. We discuss the approach to handling uncertainty in the mean growth and compare the optimal uncertainty design with the previous design.

We continue to describe how to optimize the functions using particle swarm optimization. We describe four cases operating as examples of how to use the methods. We further describe the simulated annealing algorithm and how to optimize a function in one of the same cases as the unrestricted case and compare the results. How to examine the stability of the solution is also described. This study ends with presenting and discussing the results.

## Variance as Expression of Information

It is well known that $Var\left(\hat{\theta }|\theta \right)=I{\left(\theta \right)}^{-1}$ asymptotically (Lord (1983)), where $\hat{\theta }$ is the Maximum Likelihood (ML) estimate of an examinees ability *θ* and *I*(*θ*) is the test information function. The test information function is simply the sum over items of the item information at the given ability level, that is, $I\left(\theta \right)=\sum_{i=1}^{n}I_{i}\left(\theta \right)$ where $I_{i}\left(\theta \right)=a_{i}^{2}\cdot p_{i}\left(\theta \right)\left(1-p_{i}\left(\theta \right)\right)$ for the 2PL model (Baker & Kim, 2004) parameterized as $p_{i}\left(\theta \right)=\frac{1}{1-e^{-a_{i}\left(\theta -b_{i}\right)}}$ . Here, *p*_*i*_(*θ*) is the probability of a correct response to a dichotomous item *i* with difficulty *b*_*i*_ and discrimination *a*_*i*_ for an examinee with ability *θ*.

Since the true abilities of the examinees are latent variables, they are unknown and cannot be estimated before the test is given. We will, however, assume a distribution of the abilities. Since our main interest lies in designing tests that will optimize functions of abilities of all examinees, a vector of target abilities $\theta ={\left(\theta_{1},\ldots ,\theta_{l}\right)}^{\prime }$ and associated weights ***W*** = (*w*_1_, *…*, *w*_*l*_) that corresponds to the assumed distribution of the abilities in a similar way will be used as were done by Berger (1998). This was further described in section 3.1.

If the asymptotic result was used, then one can write the variance of the estimated mean of an ability distribution as

(1) $$\text{Var}\left(\hat{\mu }\right)=\text{Var}\left(\frac{\sum_{j=1}^{N}{\hat{\theta }}_{j}}{N}\right)=\frac{1}{N^{2}}\overset{N}{\underset{j=1}{\sum }}\left(\frac{1}{I\left(\theta_{j}\right)}\right)=\frac{1}{N^{2}}\overset{N}{\underset{j=1}{\sum }}\left(\frac{1}{\sum_{i=1}^{n}I_{i}\left(\theta_{j}\right)}\right)≕\sigma_{\hat{\mu }}^{2}$$

where *N* is the size of a simple random sample of examinees taking the test and *n* is the test length, that is, the number of items. The abilities *θ*_*j*_ are unknown latent variables but can be assumed to follow some distribution, which usually is a normal one.

One interest in this study is the mean ability growth of the same population of examinees tested at two different time points. It is often customary to use the non-equivalent anchor test (NEAT) when comparing results between two tests that differ in difficulty. The abilities are vertically scaled using separate or concurrent calibration (Kolen & Brennan, 2014). We assume that the common items guarantee measuring the examinees’ abilities on the same scale as if using concurrent calibration. It is the same procedure as in Bjermo and Miller (2021), except the parameters are not estimated.

Two separate simple random samples of size *N*_1_ and *N*_2_ are drawn from the population at different time points. The distance will be denoted *d* = *μ*_2_ − *μ*_1_, where *μ*_2_ > *μ*_1_. In the same way as in equation (1), the variance of the estimate $\hat{d}$ can be written as

(2) $$\begin{array}{lll} \text{Var}\left(\hat{d}\right) & & =\text{Var}\left({\hat{\mu }}_{2}-{\hat{\mu }}_{1}\right)=\text{Var}\left(\frac{\sum_{j=1}^{N_{1}}{\hat{\theta }}_{j,2}}{N_{2}}-\frac{\sum_{j=1}^{N_{2}}{\hat{\theta }}_{j,1}}{N_{1}}\right)\\ & & =\frac{1}{N_{1}^{2}}\overset{N_{1}}{\underset{j=1}{\sum }}\text{Var}\left({\hat{\theta }}_{j,2}\right)+\frac{1}{N_{2}^{2}}\overset{N_{2}}{\underset{j=1}{\sum }}\text{Var}\left({\hat{\theta }}_{j,1}\right)=\frac{1}{N_{1}^{2}}\overset{N_{1}}{\underset{j=1}{\sum }}\frac{1}{I\left(\theta_{j,2}\right)}+\frac{1}{N_{2}^{2}}\overset{N_{2}}{\underset{j=1}{\sum }}\frac{1}{I\left(\theta_{j,1}\right)} \end{array}$$

$$=\frac{1}{N_{1}^{2}}\overset{N_{1}}{\underset{j=1}{\sum }}\frac{1}{\sum_{i=1}^{n_{1}+n_{c}}I_{i}\left(\theta_{j,1}\right)}+\frac{1}{N_{2}^{2}}\overset{N_{2}}{\underset{j=1}{\sum }}\frac{1}{\sum_{i=n_{1}+1}^{n_{1}+n_{2}+n_{c}}I_{i}\left(\theta_{j,2}\right)}≕\sigma_{\hat{d}}^{2}.$$

The number of unique items of Tests 1 and 2 is *n*_1_ and *n*_2_, and the number of common items is *n*_*c*_, which means that the test length of the two tests is *n*_1_ + *n*_*c*_ and *n*_2_ + *n*_*c*_, respectively. We will assume that *n*_1_ = *n*_2_, that is, the test lengths are the same. We also assume equal sizes of the two samples, that is, *N*_1_ = *N*_2_. In equation (2), we assume no or negligible covariance between the estimated means. This was further explained in the discussion section.

Since the estimates of the examinee’s abilities are asymptotically normal distributed (Lord (1983)), we also have

(3) $$\hat{d}∼N\left(\mu_{2}-\mu_{1},\sigma_{\hat{d}}^{2}\right)\left(\mathrm{asymptotically}\right).$$

Besides the mean ability growth, the examinee percentile growth is also often of interest in a test situation. We will, therefore, also formulate an asymptotic expression of the variance of the estimate of the *p*th percentile distance denoted ${\hat{d}}_{p}$ .

(4) $$\begin{array}{lll} \text{Var}\left({\hat{d}}_{p}\right) & & =\text{Var}\left({\hat{\theta }}_{2p}-{\hat{\theta }}_{1p}\right)=\text{Var}\left({\hat{\theta }}_{1p}\right)+\text{Var}\left({\hat{\theta }}_{2p}\right)=\frac{1}{I\left(\theta_{1p}\right)}+\frac{1}{I\left(\theta_{2p}\right)}\\ & & =\frac{1}{\sum_{i=1}^{n_{1}+n_{c}}I_{i}\left(\theta_{1p}\right)}+\frac{1}{\sum_{i=n_{1}+1}^{n_{1}+n_{2}+n_{c}}I_{i}\left(\theta_{2p}\right)}≕\sigma_{{\hat{d}}_{p}}^{2} \end{array}$$

## Method

In this section, how to use the theory in section 2 to construct the weighted functions used in the optimization will be discussed. The particle swarm and simulated annealing optimization methods will also be discussed.

### Test Design for Minimizing the Variance of Estimated Ability Growth

The main objective of this study is to optimally select items concerning difficulties that minimize the variances $\sigma_{\hat{d}}^{2}$ and $\sigma_{{\hat{d}}_{p}}^{2}$ of the estimated distances $\hat{d}$ and ${\hat{d}}_{p}$ using particle swarm optimization.

To minimize $\sigma_{\hat{d}}^{2}$ and $\sigma_{{\hat{d}}_{p}}^{2}$ , sums of inverses of the test information at different abilities *θ*_*j*_ need to be minimized. In the asymptotic results in equations (2) and (4), the true abilities of the examinees are unknown, and there are no available ability estimates. We will, however, assume that the ability distribution is known, possible from previous tests of examinees comparable to the new population of examinees.

Optimal test designs for efficient estimation of the abilities of examinees have been, among others, studied by Theunissen (1985), van der Linden and Boekkooi-Timminga (1989), Berger (1994), and Berger (1998). We will use a similar approach as Berger (1998), which was to approximate the continuous distribution of the examinees’ abilities *θ*, by using (***θ***, **W**), where $\theta ={\left(\theta_{1},\ldots ,\theta_{l}\right)}^{\prime }$ is a vector of target abilities and **W** = (*w*_1_, *…*, *w*_*l*_) is a vector of weights corresponding to a discrete distribution. If the abilities of the examinees, for example, follow a normal distribution, the most weight *w*_*j*_ will give the *θ*_*j*_ equal to the mean of the ability distribution. Since the set of possible values of *θ*_*j*_ is reduced from a continuous space to a vector of target abilities, it is customary to treat $\theta ={\left(\theta_{1},\ldots \theta_{l}\right)}^{\prime }$ as a *l*-dimensional parameter (van der Linden, 2005). The vector $\theta ={\left(\theta_{1},\ldots ,\theta_{l}\right)}^{\prime }$ now represents the population of examinees, not a multidimensional ability. This assumption creates a so-called multi-objective optimization problem since we optimize the test for the *l* different abilities simultaneously. See, for example, Chapter 4.7 in van der Linden (2005).

Since a 2PL IRT model is assumed, the test design space will be a subset of R×R where (*a*, *b*) is called a design point in that space. If we let **J** (***θ*** |**a**, **b**) = (*I* (*θ*_1_), *…*, *I* (*θ*_*t*_)), the task is now to find the items with parameter vectors (**a**, **b**) that maximizes the information vector **J** (***θ*** |**a**, **b**) at the ability levels of examinees where the ability distribution can be represented by (***θ***, **W**). This can be formalized as maximizing the function Ψ(**J** (***θ*** |**a**, **b**), **W**). It means that we would like to find a design vector (**a**, **b**) from the design space that maximizes Ψ. We will, however, keep the discrimination parameters **a** constant as was done by Berger (1998). It means that the design space will be a subset of R , and the task is now to find a design vector **b** in the design space that optimizes the function Ψ.

A common optimality criterion is the D-criterion (Atkinsson et al. (2007)), defined as maximizing the determinant of the information matrix. It is asymptotically equivalent to minimizing the covariance matrix. However, since the problem here is to estimate a set of unidimensional parameters, it is not a multidimensional problem in the true sense. Using the D-criterion can, therefore, make it more difficult to control individual objectives (van der Linden (2005)). Another common criterion, more suitable in this case, is the maximin criterion $\Psi \left(J\right.\left(\theta |a,b\right)=\underset{j}{\min}\left(I\left(\theta_{j}\right)\right)$ (Berger, 1998; van der Linden & Boekkooi-Timminga, 1989), which can be solved using linear programming.

Berger (1998) examined the optimal selection of items for estimation of the abilities *θ* with good precision for a population of examinees and presented the results for the maximin and D-criterion when the examinees’ abilities are normally or uniformly distributed. The results for the normal distribution indicated that the difficulty parameter *b* should be close to the mode of the ability distribution when the discrimination parameter *a* where kept constant and at the levels *a* = 0.5, 1, 1.5. When *a* = 2 (and larger), the optimal selection of item difficulties *b* also follows a normal distribution.

When optimizing the variance of $\hat{d}$ , being the objective here, equation (2) already provides us with a unidimensional criterion. Hence, it is not necessary to apply any other optimality criterion. We therefore propose to approach the problem by using a weighted sum applied on ***J***, where the function Ψ is defined by equation (2). We call it the multi-objective weighted sum criterion.

If the objective were to minimize the variance of the mean, one can use the function

(5) $$\Psi_{\mu }\left(J\left(\theta |a,b\right),W\right)=\overset{l}{\underset{j=1}{\sum }}w_{j}\left(\frac{1}{\sum_{i=1}^{n}I_{i}\left(\theta_{j}\right)}\right)$$

where ***b*** = (*b*_1_, *…*, *b*_*n*_) is the vector of item difficulties in the test of length *n*, and ***a*** = (*a*_1_, *…*, *a*_*n*_) is the item discrimination parameter. The weights *w*_*j*_ reflect the distribution of the examinees’ abilities.

When minimizing the variance of the difference between two means, we introduce the criterion

(6) $$\Psi_{d}\left(J\left(\theta |a,b\right),W\right)=\overset{l}{\underset{j=1}{\sum }}\left(w_{j,1}\left(\frac{1}{\sum_{i=1}^{n_{1}+n_{c}}I_{i}\left(\theta_{j,1}\right)}\right)+w_{j,2}\left(\frac{1}{\sum_{i=n_{1}+1}^{n_{1}+n_{2}+n_{c}}I_{i}\left(\theta_{j,2}\right)}\right)\right)$$

where $b=\left(b_{1},\ldots ,b_{n_{1}+n_{2}+n_{c}}\right)$ is the vector of item difficulties of Tests 1 and 2, with the common items arranged in the middle. We will assume a normal distribution of the abilities, but other choices are also possible. We discretize R and choose *l* equidistant values for the abilities *θ*_*j*,1_ and *θ*_*j*,2_ along the real line. The weights are created by letting *w*_*j*,1_ = *f* (*θ*_*j*,1_) and *w*_*j*,2_ = *f* (*θ*_*j*,2_), which are the values of the probability density functions at ability level *θ*_*j*,1_ and *θ*_*j*,2_, respectively. The weights are standardized such that $\sum_{j=1}^{l}w_{j,1}=\sum_{j=1}^{l}w_{j,2}=1$ . We will assume that the distribution of the abilities of the two populations are $N\left(\pm \frac{d}{2},1\right)$ , respectively, where *d* is the growth in mean ability. We will further assume that the item discrimination parameters ***a*** are held constant at different levels.

When minimizing the variance of the estimated percentile difference, we introduce the criterion

(7) $$\Psi_{d_{p}}\left(J\left(\theta |a,b\right),W\right)=w_{1p}\left(\frac{1}{\sum_{i=1}^{n_{1}+n_{c}}I_{i}\left(\theta_{1p}\right)}\right)+w_{2p}\left(\frac{1}{\sum_{i=n_{1}+1}^{n_{1}+n_{2}+n_{c}}I_{i}\left(\theta_{2p}\right)}\right)$$

where *p* is the *p*th percentile. The weights are now defined as *w*_1*p*_ = *f* (*θ*_*p*,1_) and *w*_2*p*_ = *f* (*θ*_*p*,2_) and are standardized such that *w*_1*p*_ + *w*_2*p*_ = 1.

Equations (6) and (7) are weighted sums, and we will use particle swarm optimization to obtain the values of the item difficulty parameters that minimize the functions in the equations.

The equations can also be combined to obtain solutions when interest lies in minimizing the variance of the estimated mean ability growth simultaneously with minimizing the estimated percentile growth for one or several percentiles. It is also possible to do this if interest lies in minimizing the variance of the estimated mean ability growth for two groups simultaneously, where the group’s abilities differ by some constant. It is possible because of the use of weights representing the distribution of the examinees’ abilities. The weights will be standardized when the equations are combined. The objective will be to find the item difficulties ***b*** that minimizes equations (6) and (7) both separately and combined.

### Uncertainty of Ability Growth

The examinees’ abilities are unknown when the test is designed. The estimates of the abilities depend on the item responses, which are unavailable before the test session. It is, however, often assumed that the examinees’ abilities follow some known distribution, for example, the normal distribution.

Even though a normal distribution is assumed, the exact mean of the distribution is unknown. Therefore, the exact growth of a population’s ability means between two grades in school is also unknown. The annual ability gain is often bigger between grades in primary school compared to middle and high school. See, for example Bloom et al. (2008), Dadey and Briggs (2012), and Tong and Kolen (2007). Average annual gains in primary school are about 0.5–1 standard deviation in lower grades and about 0.3–0.6 standard deviation in higher grades for mathematics and reading. The annual gains in middle school are about 0.2–0.3 standard deviations. In high school, the gains are even lower.

Since the mean ability gains between grades are unknown beforehand, how to obtain an optimal test design will be examined when there is uncertainty in the abilities *θ* and hence the mean *μ* and distance measure *d*. We propose to use an optimum in-average design, also called Bayesian design (Atkinsson et al., 2007) accounting for the uncertainty in *μ* and, therefore, in *d*.

**Figure 1.**
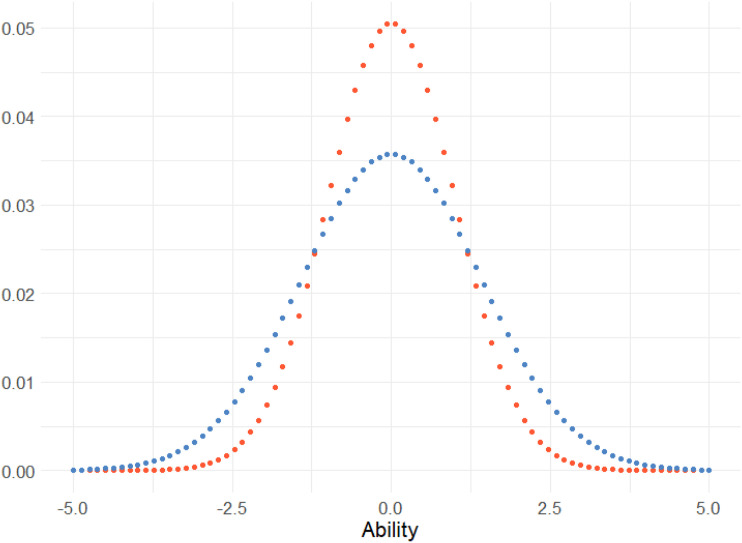
The distribution of the original weights and the optimum in-average weights (lower blue dots) when assuming that the prior is *μ* ∼ *N* (0, 1) and *l* = 80.

Firstly, this method for a single test setup was formulated where we assume a prior distribution *p*(*μ*) for *μ*. The multi-objective weighted sum we would like to optimize is, therefore, the same as in equation (5) but where the weights are now *w*_*j*_ = *∫f* (*θ*_*j*_|*x*, 1) ⋅ *f* (*x*|*μ*, *σ*^2^)*dx*, where the function *f* () is the probability density function for the normal distribution with mean and standard deviation *x*, 1 and *μ*, *σ*^2^, respectively, and the function *f* (*x*|*μ*, *σ*^2^) is the prior distribution. Since this is the same as equation (5) with different weights, it can be optimized as previously using particle swarm optimization. If we assume *μ* = 0, *σ* = 1 and integrate, the new weights will have a distribution with a heavier tail compared to the original weights, visualized in Figure 1.

Using the optimum in-average design for minimizing $\sigma_{\hat{d}}^{2}$ , we will proceed similarly as above. The weights will now be *∫f* (*θ*_*j*,1_|*x*, 1) ⋅ *f* (*x*|*μ*_1_, *σ*^2^)*dx* and *∫f* (*θ*_*j*,2_|*x*, 1) ⋅ *f* (*x*|*μ*_2_, *σ*^2^)*dx*, where *μ*_1_ and *μ*_2_ are the means that defines *d* = *μ*_2_ − *μ*_1_. We can, therefore, minimize the function in equation (6) with new weights using particle swarm optimization once again.

### Particle Swarm Optimization

Particle swarm optimization is a computational method for optimizing that intends to simulate social behavior (Kennedy & Eberhart, 1995). It is a gradient-free optimization method starting with several particles (called a swarm) randomly allocated in the search space, where the particles will move around guided by their own, and the entire particle swarm’s best-known positions. Improved positions of particles will inform the rest of the movements of the particle swarm, and the algorithm will move the particles toward the best global solution. Clerc (2006) gives a detailed description of the method.

There is no guarantee that the algorithm will find the global optimum, but Xu and Yu (2018) showed that the probability of reach depends on the transition probabilities when the number of iterations increases. As long as the series of the minimum transition probability from one state to another is divergent, the algorithm will reach the global optimal with probability one. The probability of convergence to the global optimum also depends on the choices of the hyperparameters (see Trelea, 2003). Piotrowski et al. (2020) concluded that the best results are achieved with swarm sizes of 70–500.

For the objective in this study, the discrimination parameters are kept constant to some values for all items, and we are searching for the optimal solution of the difficulty parameters, which will be the particles in the optimization procedure. For, to minimize the function in equation (5), the search space would be *n*-dimensional.

### Simulated Annealing

Since the discrimination parameter *a* and the difficulty parameter *b* come in pairs for every item, they are related. Section 3.1 describes the scenario where we keep the discrimination parameter *a* constant at different levels. For a more realistic solution, we need to include the *a*-parameter in the optimization process. But including the *a*-parameter without any constraint would create a solution where all the values of the *a*-parameter vector would equal the upper bound. It follows from the information function for the 2PL model. Therefore, an item pool with estimated calibrated items capturing the natural relations between the *a*- and *b*-parameters was created.

The particle swarm optimization method is suitable when the search space is continuous, and it is therefore not possible to use for selecting items from an item pool to optimize Function 6, which is a discrete optimization problem. Hence, simulated annealing was used as the optimization algorithm for selecting items from the pool.

Simulated annealing (Kirkpatrick et al., 1992) is an optimization technique for approximating the global optimum of a function. It is often used for discrete search spaces, making it a good option when optimally selecting items from an item pool. The name simulated annealing originates from the physical process of heating a material and then lowering the temperature in a controlled way to alter its physical properties. See, for example, Kirkpatrick et al. (1992) and Reeves (1993) for a detailed description of the method.

The method starts with a random solution and then chooses a neighborhood solution of the current solution, which is accepted with some probability. The probability of accepting the new solution depends on the so-called energy of the solutions and a parameter called the temperature. The algorithm will always accept the new solution if it improves the function value of the solution, otherwise, it will accept the new solution with the acceptance probability. The temperature will gradually decrease during the iterations making it less probable to accept a worse solution as time passes. It makes the algorithm explore a wider range of solutions, even if they are worse, earlier in the process. Besides the choice of the initial temperature and definition of the neighborhood, the algorithm needs a cooling schedule and temperature reduction function. The cooling schedule is a parameter that decides the number of iterations before every temperature reduction, and the reduction function quantifies the magnitude of the temperature decrease in every cooling cycle, which we will call an epoch.

The definition of the neighborhood, the starting temperature, the temperature reduction function, the number of epochs, the number of iterations in every epoch, and the stopping criteria are determined in advance.

The neighborhood is defined such that the optimal solution is reachable from any starting solution (Anily & Federgruen, 1987). The initial temperature has to be large enough to produce a final solution independent of the starting solution (Reeves, 1993). To ensure convergence to a global optimum or a local solution approximately optimal, theory suggests that the algorithm should be allowed to move close to its stationary distribution at every temperature and that the temperature will gradually decrease to zero. This may be achieved by a large number of iterations at a few temperatures (Reeves, 1993). The stopping criteria can be defined as when the number of iterations reaches a pre-determined number of counts (Park & Kim, 1998).

Algorithm 1 shows the algorithm implemented in the statistical software R.


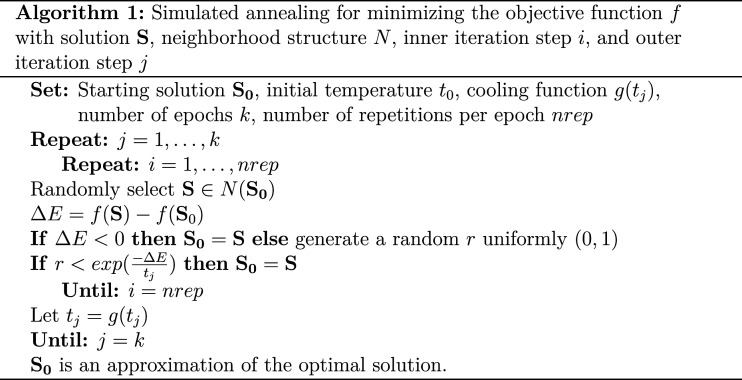


## Performing the Optimization

This section describes the particle swarm optimization setup and how they conduct it in four cases. We also describe how to perform it when introducing uncertainty in the ability growth. The section also describes the simulated annealing setup when selecting items from an item pool. At last, we present how to examine the stability of the particle swarm solution.

### Optimization Setup

The functions presented in section 3.1 and 3.2 are used to find the optimal selection of item difficulties of the test by applying the particle swarm optimization method. Functions 6 and 7 are created as functions of the difficulty parameter vector **b**. The functions also include the weights defined in Section 3, the number of weights *l*, the distance *d*, and the discrimination parameter **a**.

The functions were defined as multi-objective weighted sums as proposed by Yang (2014) ch. 14. The function psoptim () was used (Ciupke, 2022) from the package pso in the statistical software R.

The optimal values will be determined for the item difficulty parameters $b_{1},b_{2},\ldots ,b_{n_{1}}$ belonging to Test 1, $b_{n_{1}+1},\ldots ,b_{n_{c}}$ belonging to the common items, and $b_{n_{c}+1},\ldots ,b_{n_{2}}$ belonging to Test 2. The item difficulty parameters are now the particles in the optimization process.

The search space during the optimization will now have the same dimension as the test length *n*_1_ + *n*_2_ + *n*_*c*_ = 75, and the lower and upper bounds are set to −6, 6. Performing the particle swarm optimization will result in an optimal solution of $b_{1},b_{2},\ldots ,b_{n_{1}},b_{n_{1}+1},\ldots ,b_{n_{c}},b_{n_{c}+1},\ldots ,b_{n_{2}}$ .

The hyperparameters in the algorithm are tuned as follows: proportion informed = 0.2, the particle swarm size = 200, and the convergence criterion = 1 ⋅ 10^−10^. Different values were tested, and the resulting solutions with the given parameters are very similar under repetition, indicating a stable solution. This is further explored in section 4.5.

### Optimal Designs for Four Cases

The optimal values of the item difficulty parameters will be determined as described in Section 4.1 for four different cases. Firstly, we will minimize the variance of the estimated mean difference. Secondly, we will minimize the variance of the estimated mean difference simultaneously as minimizing the variance of the estimated percentile difference for the 25th and 75th percentile. Thirdly, we will consider minimizing the variance of the estimated percentile difference for percentiles 25, 50, and 75. Lastly, the variance for the mean difference for two groups will be minimized with an ability difference of 0.25. This equivalent to $\theta_{j_{2},1}=\theta_{j_{1},1}+0.25$ for every *j*_1_ and *j*_2_ in the two groups. The weights can of course be modified to fit a real testing situation depending on the objective.

For the first case, the weights consist of *l* = 100 equidistant values from the probability density functions from the two normal distributions $N\left(\pm \frac{d}{2},1\right)$ .

For the second case, the same weights are used as in the first case but also add weights for the percentiles. Once again with *l* = 100. In the third case, use the weights for the percentiles, which means that *l* = 6. For the fourth case, the weights will be as in the first case with *l* = 100 but with a 0.25 ability shift between groups.

The particles in the optimization process are the difficulty parameters even though the discrimination parameters also contribute to the Fisher information. The optimization will, therefore, be performed when the values of the discrimination parameters are held constant at different levels *a* = 0.5, 1, 1.5, 2. It means that we perform the optimization four times for every value *d* = 0.5, 1, 1.5, 2, 2.5 resulting in 4 ⋅ 5 = 20 optimization runs per case. Since *a* strongly influences the information, we can observe how the results vary with different values of *a* in plots in Section 5.

### Optimal Sampling from an Item Pool

An item pool was created from item parameters of all 2PL items in Grade 8 mathematics from the Timss 2011, 2015, and 2019 administrations (International Study Center, 2022). The item pool consists of 248 unique 2PL items with estimated item parameters. The mean was subtracted from the difficulty parameter values *b*, creating new parameters with mean zero comparable to the parameters in the particle swarm method.

The means of the *a*- and *b*-parameters are then 1.14 and 0, and they span between 0.40 and 1.90, and −1.56 and 1.60, respectively.

We define the neighborhood as the set of solutions obtained by swapping one item in the current sample with one item of the remaining item pool. This simple neighborhood guarantees that the optimal solution is reachable (Anily & Federgruen, 1987) from any starting solution.

The variance of the estimated mean difference was used, equation (6), as the objective function to minimize. We produced a random starting solution of 75 items from the pool and set the starting temperature to *t*_0_ = 100 to guarantee an acceptance probability close to 1 for “uphill” moves in the first epochs. The cooling function where defined as *g* (*t*_*j*_) = *αt*_*j*_ where *α* = 0.8. The number of iterations at every temperature is *nrep* = 5000, big enough for the algorithm to explore the solution space at every epoch. The number of epochs is 100. With initial temperature *t*_0_ = 100 and *α* = 0.8, the temperature decreases to near zero at the end of the algorithm. We also run a greedy algorithm with 100,000 iterations with the solution as a starting value only accepting improvements. No further improvements were observed, implying an approximative optimal solution.

The solution is now in the form of 75 items that produced the lowest objective function value when the simulated annealing algorithm stopped. We make the optimization for *d* = 0.5, 1, 1.5, 2, 2.5 and present the plot of the item difficulties for the selected items in Section 5.2. Scatter plots of the items are also presented in the final solutions for all values of *d*.

### Uncertainty About the Mean Ability Growth

To test the approach with optimum in-average weights, we will compare values of equation (2) for the optimization with original weights to the optimization with optimum in-average weights.

To examine the optimum in-average approach, let the priors be $\mu_{1}∼N\left(-\frac{d}{2},0.2\right)$ and $\mu_{2}∼N\left(\frac{d}{2},0.2\right)$ . We further assume that every item discrimination parameter is randomly sampled as *a*_*i*_ ∼lognorm (0, 0.25), *i* = 1, …, *n*_1_ + *n*_2_ + *n*_*c*_ (see Section 4.3).

Particle swarm optimization with the optimum in-average weights was used in equation (6) to obtain the optimal design for the item difficulties of a test with *n*_1_ = *n*_2_ = 30, *n*_*c*_ = 15, and when *d* = 1. Then we compute $\sigma_{\hat{d}}^{O}/\sigma_{\hat{d}}^{A}$ for the values *d* = (0.4, 0.6, …, 1.4, 1.6), where $\sigma_{\hat{d}}^{O}$ is calculated under the optimal design for *d* = 1 and $\sigma_{\hat{d}}^{A}$ under the optimum in-average design. If the ratio exceeds 1, the optimum in-average design is better. The results are presented as boxplots so that all ratios for every iteration are visible.

### Stable Solution

The function psoptim() (Ciupke, 2022) in R uses random starting points by default. Therefore, we performed an optimization 50 times and presented the mean and standard deviation of the solution for every value of *b*.

The optimization using *a* = 1.5 and *d* = 2.5 was repeated and saved the resulting solution of the 75 *b*-values in a vector for every repetition. The mean and standard deviation were then calculated for every component in the vector **b**, resulting in 75 mean and standard deviation values, respectively. The results were presented as plots in the result section.

## Results

### Optimal Designs for all Cases

**Figure 2.**
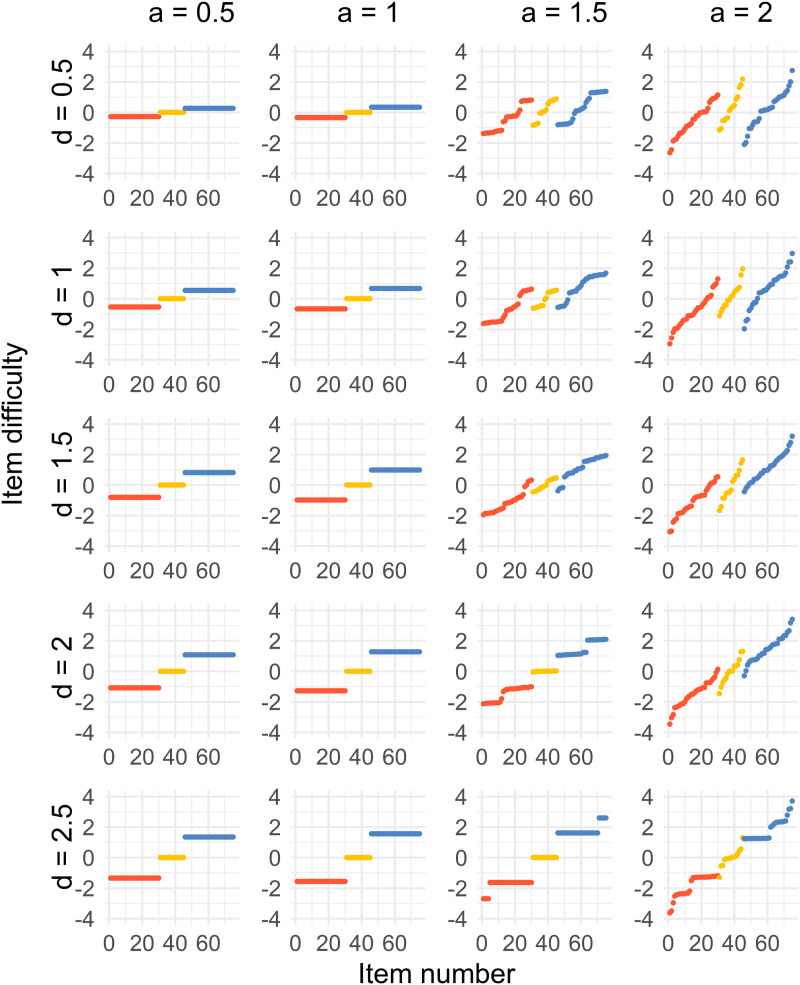
Distribution of sorted item difficulty parameters for the Case 1 optimal design. The columns represent the item discrimination *a* = 0.5, 1, 1.5, 2 and the rows represent the distances *d* = 0.5, 1, 1.5, 2, 2.5. The red dots (left), yellow dots (middle), and blue dots (right) represent the Test 1 items, common items, and Test 2 items, respectively.

**Figure 3.**
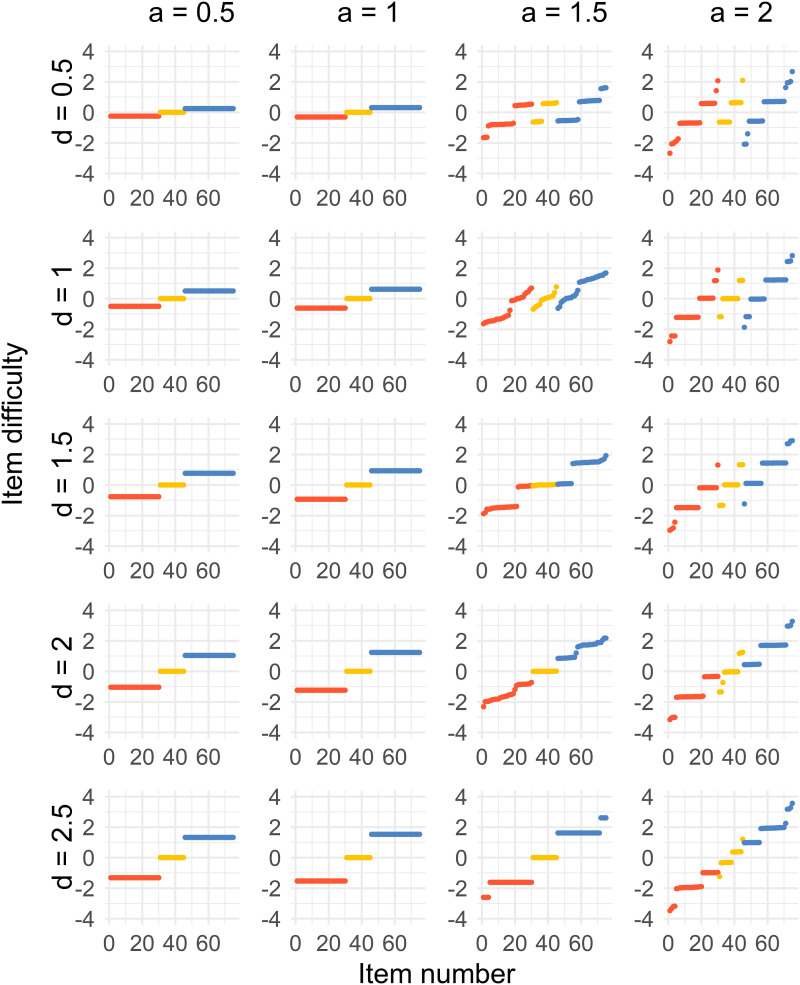
Distribution of sorted item difficulty parameters for the Case 2 optimal design. The columns represent the item discrimination *a* = 0.5, 1, 1.5, 2 and the rows represent the distances *d* = 0.5, 1, 1.5, 2, 2.5. The red dots (left), yellow dots (middle), and blue dots (right) represent the Test 1 items, common items, and Test 2 items, respectively.

**Figure 4.**
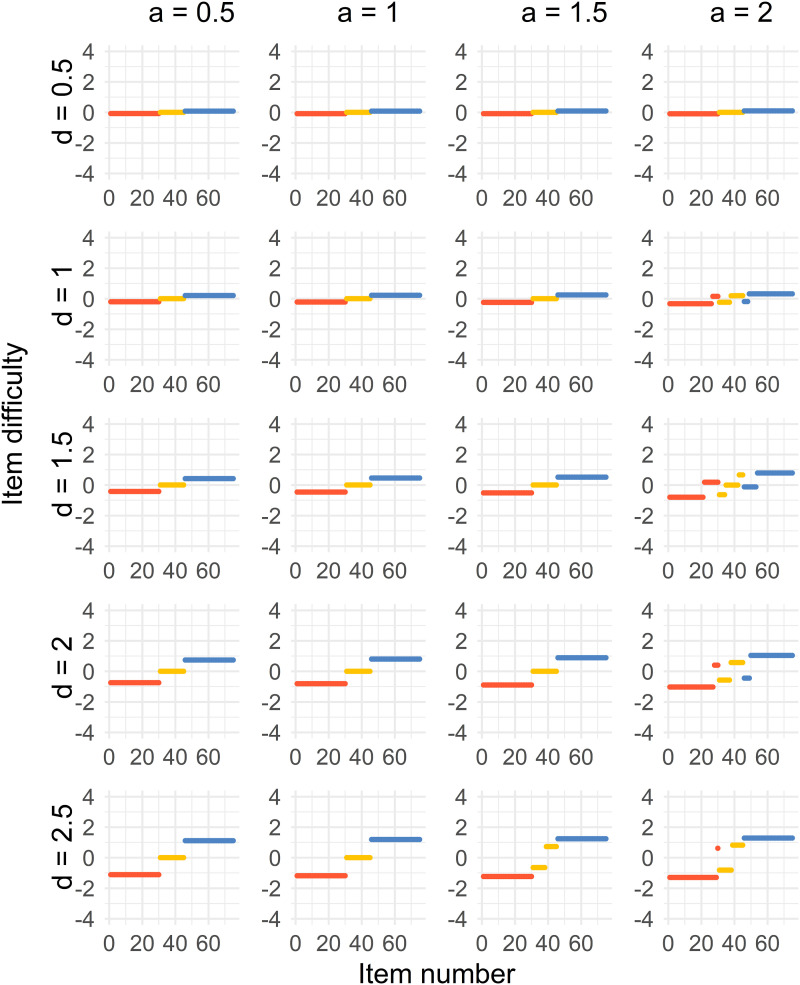
Distribution of sorted item difficulty parameters for the Case 3 optimal design. The columns represent the item discrimination *a* = 0.5, 1, 1.5, 2 and the rows represent the distances *d* = 0.5, 1, 1.5, 2, 2.5. The red dots (left), yellow dots (middle), and blue dots (right) represent the Test 1 items, common items, and Test 2 items, respectively.

**Figure 5.**
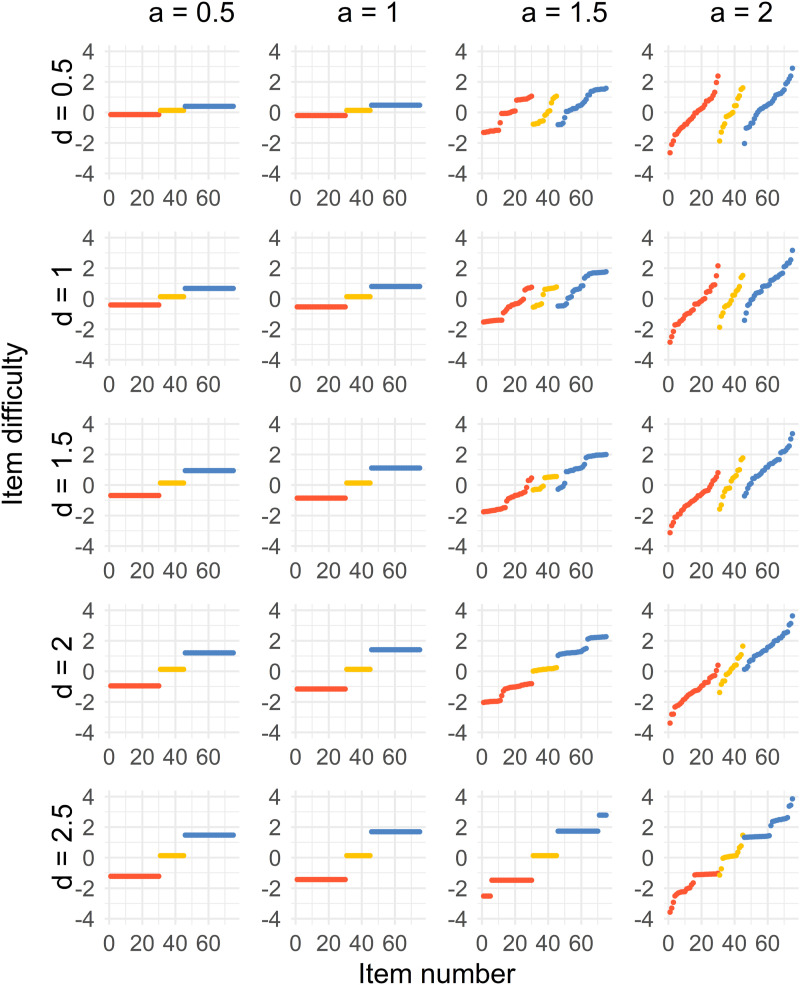
Distribution of sorted item difficulty parameters for the Case 4 optimal design. The columns represent the item discrimination *a* = 0.5, 1, 1.5, 2 and the rows represent the distances *d* = 0.5, 1, 1.5, 2, 2.5. The red dots (left), yellow dots (middle), and blue dots (right) represent the Test 1 items, common items, and Test 2 items, respectively.

The results for all four cases can be seen in Figures 2–5. There is one figure per case, and every figure displays the solution for different values of *a* and values of *d*. Generally, the spread of the difficulty parameters depends on *d* and *a*.

In the Figure 2, we see that a three-point solution is optimal for low values of the discrimination parameter *a* < 1.5. The difficulties of the common items are close to *b* = 0, and the Tests 1 and 2 item difficulties are at equal distances from the common items, with increasing distances with higher values of *d*.

For higher values, *a* ≥ 1.5, the optimal solution is more spread out. It is true for the common, Test 1 and Test 2 items. For increasing *d*, the optimal solution when *a* ≥ 1.5 approaches a point solution by increasing *d*. For example, the optimal solution is a five-point solution when *a* = 1.5.

For the second case, where *a* < 1.5, a three-point solution is the optimal solution for all values of *d*. The difficulties of the common items are close to *b* = 0, and the Test 1 and 2 item difficulties are on equal distances from the common items, where the distance increases with higher values of *d*.

When *a* ≥ 1.5, the optimal solution is more spread out but different compared to Case 1. Some of the optimal solutions get more discretized when *a* = 2 compared to when *a* = 1.5, which is visible in the most right column of Figure 3. As for Case 1, the optimal solution approaches a point solution by increasing *d* when *a* ≥ 1.5.

Case 3 (Figure 4) provides us with a three-point optimal solution for most of the combinations of *d* and *a*, where the common items are close to *b* = 0, and the Tests 1 and 2 item difficulties are on equal distances from the common items, where the distance increases with higher values of *d* and *a*. The only exceptions are when *a* = 2 and *d* = 1, 1.5, 2, where the unique and common items split up. For *a* = 1.5, 2 and *d* = 2.5, the solution split up only for the common items.

Case 4 (Figure 5) gives us a similar solution to Case 1 with a shift in the solution, which is unsurprising since the groups are just using the same weights as the other group with a difference of 0.25 in ability.

### Optimal Items from the Item Pool

**Figure 6.**
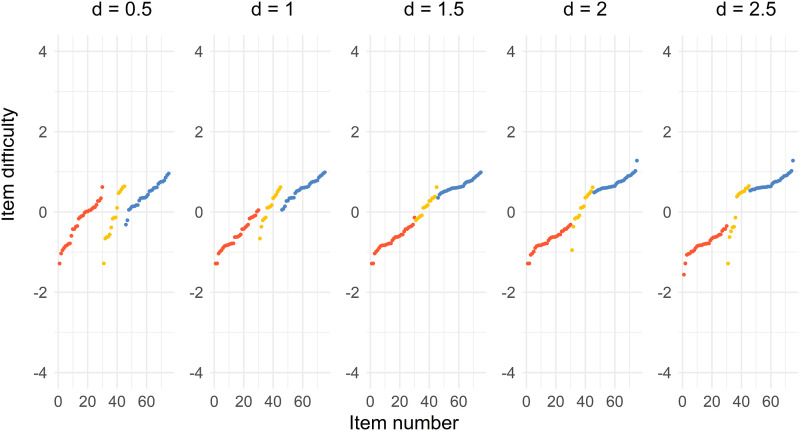
Distribution of sorted item difficulty parameters for the item pool optimal design. The columns represent the distances *d* = 0.5, 1, 1.5, 2, 2.5. The red dots (left), yellow dots (middle), and blue dots (right) represent the Test 1 items, common items, and Test 2 items, respectively.

Figure 6 shows that the optimal solution for choosing items from an item pool is somewhat similar to the Case 1 solution when *a* = 2 presented in Figure 2. The solutions for the Test 1, the common, and Test 2 items are separated when *d* = 0.5 and *d* = 1. The solutions merge into one single distribution, especially for *d* = 1.5, but also for *d* = 2. The common items are located in the middle of the Test 1 and Test 2 items. The solutions tend to form two separate distributions when *d* = 2.5, where half of the common items are close to the Test 1 items and the other half is close to the Test 2 items. The spread of the difficulty parameter is also less compared to the Case 1 solution, because the *b*-parameter in the pool spans −1.56 and 1.60.

**Figure 7.**
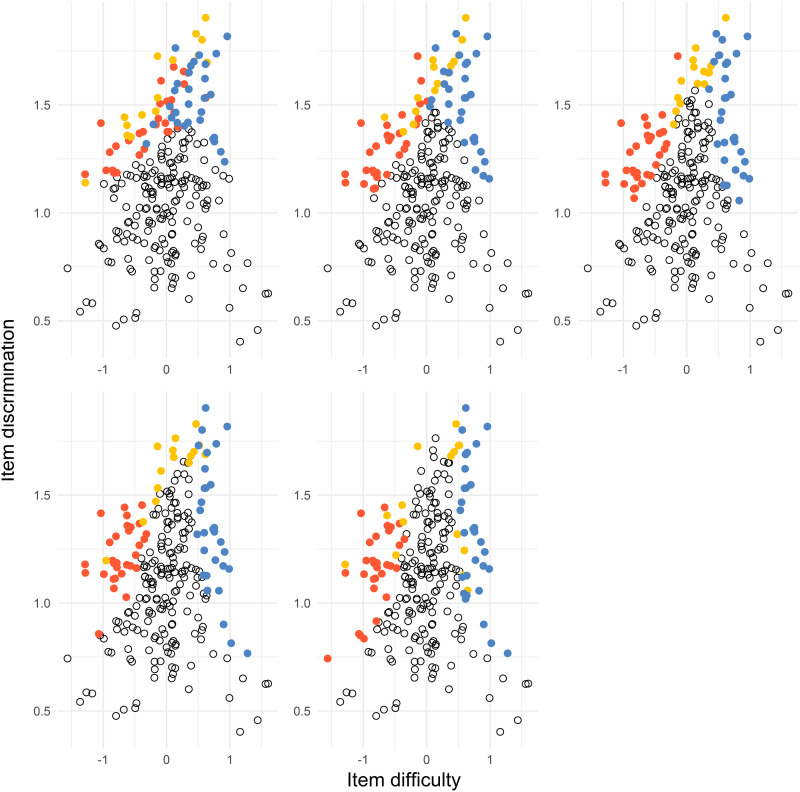
The item pool items where every dot represent an item. The subplots represent the distances *d* = 0.5, 1, 1.5, 2, 2.5 from top left to bottom right. The red dots (left), yellow dots (middle), and blue dots (right) represent the Test 1 items, common items, and Test 2 items, respectively. The black dots represent the remaining items in the item pool.

In Figure 7, we can see that the item in the solution includes the easiest items with the largest discrimination (Test 1 items), the most difficult items with the largest discrimination (Test 2 items), and common items with the largest discrimination and difficulty depending on the value of *d*.

### Optimal Design for the Uncertainty of Ability Growth

**Figure 8.**
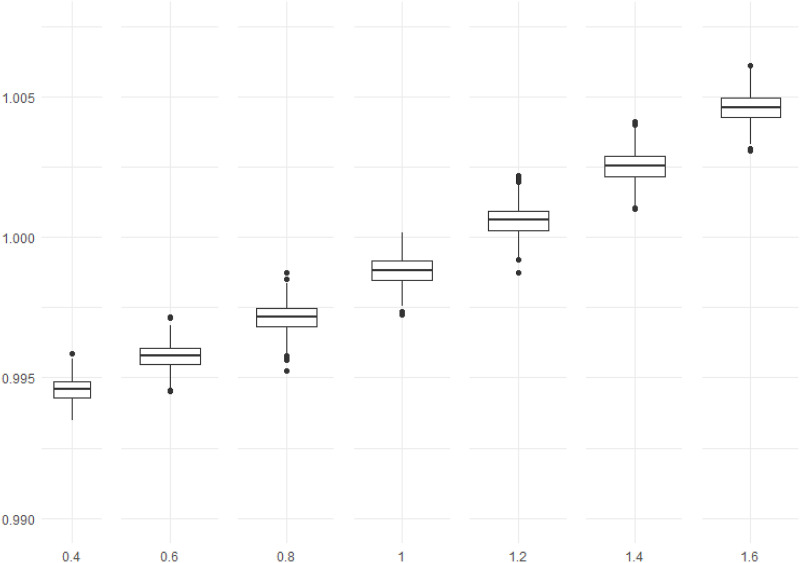
Box plots of the 1000 calculated ratios $\frac{\sigma_{\hat{d}}^{O}}{\sigma_{\hat{d}}^{A}}$ where O means the ordinary optimal design and A the optimum in-average design. The *x*-axis shows the true values of *d*.

Figure 8 shows that the optimum in-average design is better for values larger than about *d* = 1. We can interpret this as to prefer the optimum in-average design for true values larger than *d*. The results here depend on the chosen prior distribution, especially the variance. It could be explored further which values are optimal.

The calculated ratios in Figure 8 are very close to 1 for all values of the true distance *d*, meaning that the effect is not severe for using the optimum in-average design. Besides the chosen prior, different values of *a* and *d* will also affect the results.

### Stable Solution

**Figure 9.**
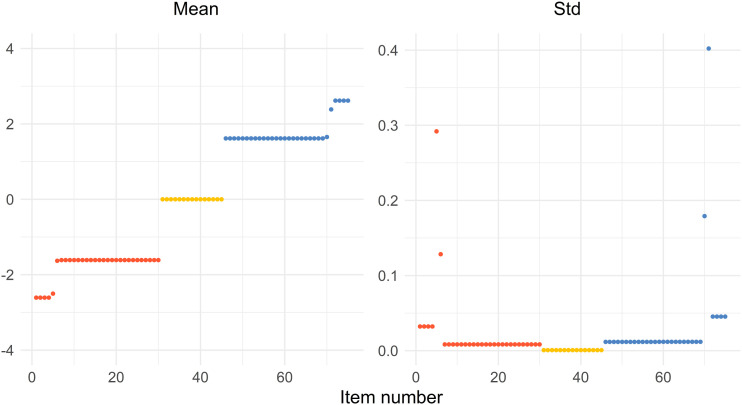
The distribution of the means (left) and the standard deviations (right) of sorted difficulty parameters from 50 optimizations. The red dots (left), yellow dots (middle), and blue dots (right) represent the Test 1 items, common items, and Test 2 items, respectively.

Figure 9 shows the mean and standard deviation of 50 optimizations for every value of the difficulty parameter *b* when *d* = 2.5 and *a* = 1.5. The mean is very similar to configuration *d* = 2.5 and *a* = 1.5 in Figure 2 except for some points near the edges of the outermost and next to the outermost group of solutions. That is confirmed by the right part of the figure displaying the standard deviations. The standard deviation is low except for those points mentioned above, that is, near the edges. It indicates that it is fair to claim stable solutions using particle swarm optimization.

## Discussion

The main objective of this study was to demonstrate how to use the test information function to optimally allocate the item difficulty parameters in a test design situation where the focus lies in measuring ability growth. Optimal allocation means the choice of item difficulties minimizing the variance of the estimated difference in means and percentiles between two populations. The method represents growth in mean ability and percentile growth between populations of examinees in different school grades.

We presented the weighted functions for the variance of the estimated ability mean growth and *p*th percentile growth, and one can choose the weights to represent the distribution of the examinees’ abilities, making it a flexible method. An optimal solution for the item difficulty parameters was obtained by applying a particle swarm optimization algorithm. The algorithm always converges to similar solutions under repetition, which indicates that it is unlikely with several different local optima. Even though we cannot be sure of a global optimum, we achieve a low variance.

The solutions for four different cases are displayed. In Case 1, the focus is on mean ability growth. Case 2 adds the 25th and 75th percentile difference to Case 1. Case 3 only considers the 25th, 50th, and 75th percentile differences. And at last, Case 4 considers two groups with the same ability distributions but with a shift in the distribution.

For all the cases, the results varied with different values for the distance *d* and the value of the discrimination parameter *a*. For all cases, when *a* < 1.5, the difficulties for the common items are close to zero, and the solutions shift by a constant for Case 4. The Test 1 and Test 2 items difficulties are allocated at almost the same values but on different sides of the common items with an equal distance. When *a* > 1, the solution of the item difficulties starts to spread out even if it differs between different cases and with different values of *d*. One reason for the difference is the square of the discrimination parameter *a* in the item information function. When *a* is smaller, the items with parameter *b* near where the majority of the weights of the abilities are dominant. Whereas when *a* is larger, items with *b*-values further out will influence the result more. For increasing *d*, the weights for the two distributions will be more separated, which affects the results so that the solution tends to be a point solution again for larger values of *a*.

The result by Berger (1998) suggested that for normally distributed abilities, the item difficulty parameters are close to the mode or median when using the maximin or D-criterion for *a* < 2. The result applies to the one-test situation, which differs from the two-test situation in this study. However, the results that item difficulties are close to single point value for smaller values of *a* is similar to the results in Berger (1998) even though the optimality criteria are different.

The result discussed above refers to the unrestricted situation where any value of the difficulty parameter *b*, within the bounds, is allowed. In a testing situation, the discrimination parameter *a* and difficulty parameter *b* are paired and related since they belong to the same item. It suggests including the *a*-parameter in the optimization. Doing so in an unrestricted scenario would end in an optimal solution where the *a*-parameters are as large as possible within the pre-determined bounds.

For comparison, we performed a discrete optimization by selecting items from an item pool. An item pool from estimated 2PL items from released Timss items was created. We then used the simulated annealing algorithm that produced an approximately optimal design. The results indicate similarities with the solutions for the Case 1 optimization using particle swarm optimization where *a* = 2. It is clearly visible that the items with the largest values of the *a*-parameters end up in the optimal solution.

With the help of the results from the unrestricted cases using particle swarm optimization, this study can advise which items to include in an item pool and how to choose starting values for a combinatorial optimization problem, such as the simulated annealing example above. A wise-chosen starting value could speed up the algorithm in cases where it is of interest. How to improve the simulated annealing process can be a future research topic, for example, how to optimally choose the starting temperature and how to define a neighborhood in a wiser way.

A way to handle the uncertainty in mean ability growth *d* was also developed by introducing a prior distribution. We integrated the true criterion (i.e., the weights) over a prior distribution and performed the particle swarm optimization again. The results show that the optimum in the average method performed better for values above the true value even though resulting in small improvements. It means that the method would be applicable when there is a suspicion that the true mean growth distance is larger than during previous test occasions, but it is not clear by how much. Since the difference when using the optimum in-average approach is small, we conclude that the original approach is robust against uncertainties in the growth of mean ability between school grades.

We assumed that the two compared groups were different random samples from the same population at different time points. One could argue that there is a sample dependence, demanding including a covariance in equations (2) and (4). However, since we assume that the samples are different, we can assume that the covariance is small enough to be ignored. We would have to consider the covariance if we assumed the same sample taking the test at two different time points. In such a case, the proposed NEAT design with common items would not be suitable since the examinees would be exposed to the common items twice. If individual growth is of interest as well, there will be a need for some other design that does not use common items. That is a task for further research.

The results are based on computations using individual abilities. A multivariate approach might be suitable since the main interest here is the variance of differences between group means and percentiles. An advantage of the approach used in this study is that we are not limited to certain ability distributions needed in a multivariate approach. It means that weights can be used by representing any distribution of abilities. Low- and high-achieving examinees likely have different ability growth patterns between grades. Therefore, this method can also be used to examine growth where the variance differs between two points in time.

Other constraints, such as the content of an item and time spent on answering an item, could also be of interest to include in the optimization procedure. It could be included using constrained particle swarm optimization based on the method suggested by Parsopoulos and Vrahatis (2002). How to exactly do this can be a subject of future research.
